# Intelligent response hydrogel based on choline phosphorylated chitosan programmed repair of infected wounds

**DOI:** 10.1093/rb/rbag022

**Published:** 2026-02-19

**Authors:** Min Lu, Tong Sun, Chongxu Tang, Xinmei Zhang, Shuyue Hao, Mei Yang, Qiangwei Xin, Zhongqiang Zhu, Mingming Ding, Jie Weng, Zhiqiang Li, Xingyu Chen, Jianshu Li

**Affiliations:** Institute of Biomedical Engineering, College of Medicine, Southwest Jiaotong University, Chengdu 610031, China; College of Polymer Science and Engineering, National Key Laboratory of Advanced Polymer Materials, Sichuan University, Chengdu 610065, China; Institute of Biomedical Engineering, College of Medicine, Southwest Jiaotong University, Chengdu 610031, China; Institute of Biomedical Engineering, College of Medicine, Southwest Jiaotong University, Chengdu 610031, China; College of Life Sciences and Engineering, Southwest Jiaotong University, Chengdu 610031, China; Department of Geriatrics, Hospital of Chengdu University of Traditional Chinese Medicine, Chengdu 610075, China; College of Polymer Science and Engineering, National Key Laboratory of Advanced Polymer Materials, Sichuan University, Chengdu 610065, China; College of Polymer Science and Engineering, National Key Laboratory of Advanced Polymer Materials, Sichuan University, Chengdu 610065, China; College of Polymer Science and Engineering, National Key Laboratory of Advanced Polymer Materials, Sichuan University, Chengdu 610065, China; Institute of Biomedical Engineering, College of Medicine, Southwest Jiaotong University, Chengdu 610031, China; Institute of Biomedical Engineering, College of Medicine, Southwest Jiaotong University, Chengdu 610031, China; Department of Orthopedics, The General Hospital of Western Theater Command, College of Medicine, Southwest Jiaotong University, Chengdu 610083, China; Institute of Biomedical Engineering, College of Medicine, Southwest Jiaotong University, Chengdu 610031, China; College of Polymer Science and Engineering, National Key Laboratory of Advanced Polymer Materials, Sichuan University, Chengdu 610065, China

**Keywords:** hydrogel, stimuli-responsive, antibacterial, angiogenesis, wound healing

## Abstract

The healing of infected wounds is a formidable clinical challenge that demands advanced biomaterials capable of simultaneously eradicating pathogens and orchestrating the complex regenerative process. Herein, we engineered an immunomodulatory and angiogenic multifunctional hydrogel dressing by integrating choline phosphorylated chitosan (CS-MCP) with tetracycline-loaded polydopamine nanoparticles (PDA@TH NPs) for the programmed healing of infected wounds. This composite hydrogel demonstrates on-demand, spatiotemporally controlled therapeutic responses. Under near-infrared (NIR) irradiation, it enables synergistic bactericidal activity through photothermal effects and triggered antibiotic release. Moreover, the system exhibits pH-responsive drug release behavior, specifically targeting the acidic microenvironment of infected tissues. Beyond its potent antibacterial function, the hydrogel actively promotes regenerative processes. *In vitro*, CS-MCP markedly enhanced the adhesion, proliferation and tube formation of human umbilical vein endothelial cells, demonstrating potent pro-angiogenic effects. Furthermore, the polydopamine nanoparticles effectively scavenged reactive oxygen species (ROS), attenuating oxidative stress and inducing M2 macrophage polarization to foster an immunoregulatory microenvironment conducive to tissue repair. In a rat model of *Staphylococcus aureus*-infected full-thickness skin defects, the hydrogel significantly accelerated wound healing by comprehensively modulating the entire regeneration cascade: eliminating infection, mitigating inflammation, promoting angiogenesis, and enhancing collagen deposition. This study presents a novel immune-engaging and pro-regenerative strategy, representing a highly promising platform for the treatment of refractory infected wounds.

## Graphical abstract

**Graphical Abstract:**
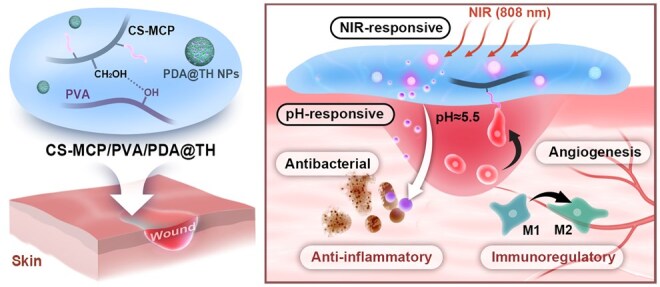

## Introduction

As the primary barrier of the body, the skin is indispensable for homeostasis, primarily by safeguarding against dehydration and a myriad of external aggressors to ensure the integrity of internal tissues [1]. However, when the skin is damaged, it becomes susceptible to microbial invasion by bacteria such as *Escherichia coli*, *Staphylococcus aureus* [2, 3]. These bacteria can colonize the wound site and cause infections, leading to excessive oxidative stress, poor neuropathy and angiopathy, which impede the healing process of the wound [4–6]. Previous studies have demonstrated that the wound healing process typically involves four overlapping stages: hemostasis, inflammation, cell proliferation (granulation tissue generation) and maturity (tissue remodeling) [7]. Unfortunately, the majority of wound dressings currently used in clinical practice only provide benefits in specific stages of wound healing, e.g., bandages and gauze are mainly used to maintain wound dryness. However, a warm and moist wound environment is crucial as it facilitates the healing process. An ideal wound healing material should play a programmed role in all these stages, including antibacterial, anti-inflammatory, immunoregulatory, angiogenic and tissue regenerative [8].

Hydrogel wound dressings have been developed as superior alternatives to other dressings [9, 10], providing a moist environment, absorbing exudate from the wound, acting as a key barrier against bacterial invasion and preventing secondary injury when changing dressings [11]. Recently, hydrogels that deliver local antibacterial drugs have garnered interest in the field. These hydrogels enable the prolonged retention of antimicrobial agents and reduce uncontrolled diffusion into surrounding tissues. Hydrogels responsive to exogenous stimuli or endogenous stimuli can intelligently control drug release [12–21]. Duan *et al.* fabricated NIR-responsive carrier-free nanoparticles (BI NPs) composed of berberine hydrochloride (BH, a phytochemical) and indocyanine green (ICG, a photosensitizer) for synergistic antibacterial therapy of infected wounds [22].

Angiogenesis is essential for wound healing because it facilitates the delivery of nutrients [23–25], which promotes regeneration and repair of wound tissue. Chitosan (CS) is a natural polysaccharide material that possesses biocompatibility, non-toxicity, biodegradability and bioactivity [26]. Choline phospholipid (CP) is a novel water-soluble zwitterion with a reverse chemical structure and charge distribution of that of the phosphatidylcholine (PC) molecule on cell membranes. This allows CP to form specific electrostatic interactions with PC [27]. In our previous work, we modified the surfaces of biomaterials with CP monomers or 2-methacryloyloxyethyl choline phosphate (MCP) polymers, resulting in modified materials that effectively promote cell adhesion and proliferation [28–34]. Additionally, we synthesized a novel CS derivative (CS-MCP) by grafting MCP molecules onto CS via the Michael addition reaction, with significantly improved water solubility and antibacterial activity due to the zwitterionic and anti-fouling properties of MCP. Interestingly, CS-MCP also exhibited superior cell proliferation characteristics when compared to unmodified CS [35–37]. Therefore, we hypothesize that the synergistic integration of choline phosphorylated CS (CS-MCP) and tetracycline-loaded polydopamine nanoparticles (PDA@TH NPs) within a multifunctional hydrogel will enable programmed, multi-stage wound healing in infected skin defects, through: pH-responsive and NIR-triggered antibacterial action for infection control, CS-MCP-mediated angiogenesis for tissue regeneration and polydopamine nanoparticles (PDA NPs) driven immunoregulation via ROS scavenging and M2 macrophage polarization.

**Figure 1:**
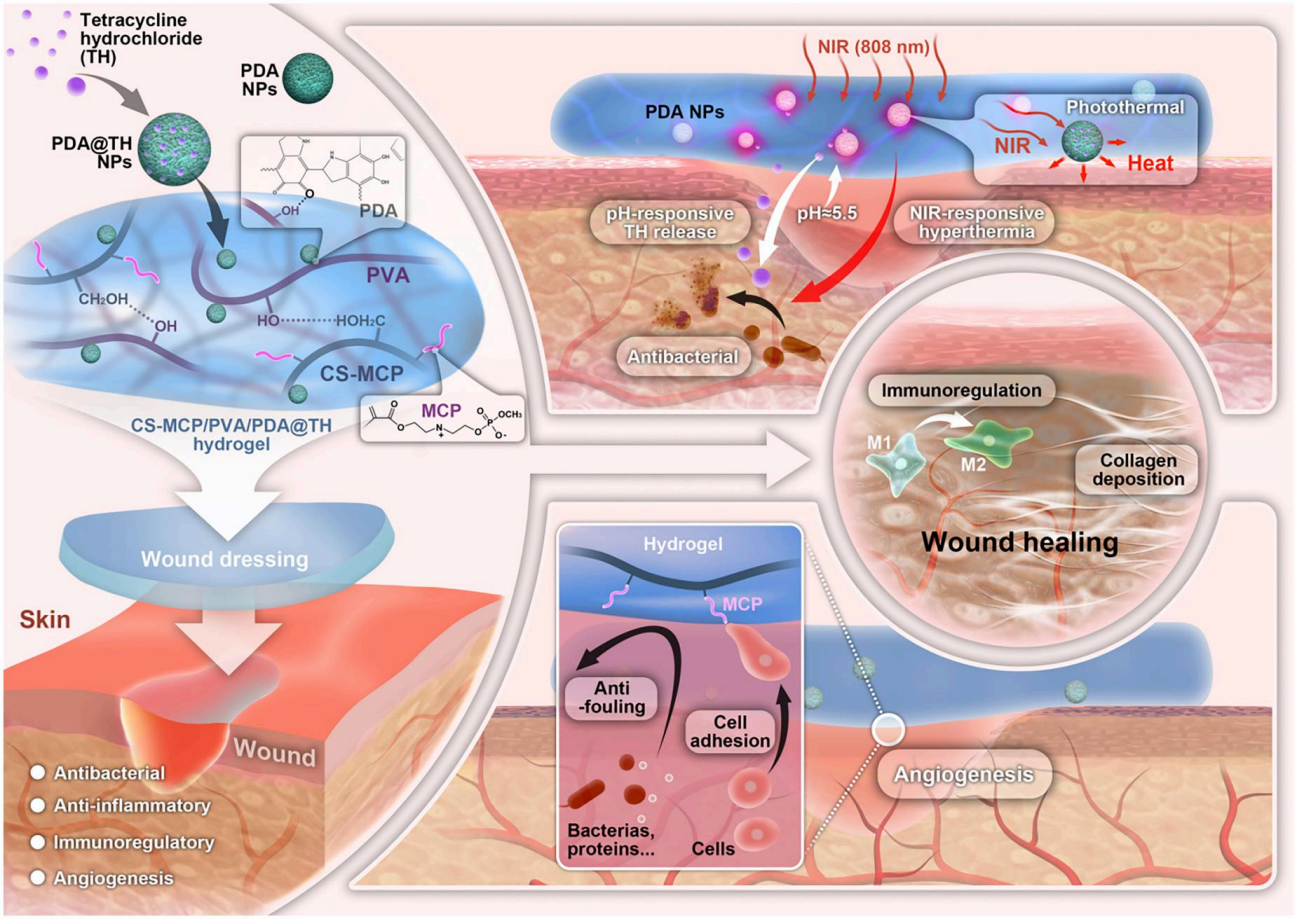
Schematic illustration of pH/NIR dual-responsive hydrogel with programmed antibacterial, anti-inflammatory, immunoregulatory and angiogenic properties for the repair of infected skin wounds.

In this study, we propose an intelligent-responsive hydrogel that can promote the healing of infected wounds in a programmed manner (Figure 1). The hydrogel matrix is composed of CS-MCP and polyvinyl alcohol (PVA) through hydrogen bonding, and then tetracycline hydrochloride (TH)-loaded PDA NPs can be cross-linked with CS-MCP/PVA hydrogel via quinone groups with amino and hydroxyl groups. Under acidic conditions, PDA NPs and TH can achieve responsive release from the hydrogel matrix. In addition to photothermal antibacterial capability, PDA NPs possess additional therapeutic functionalities, including scavenging reactive oxygen species (ROS) to alleviate oxidative stress and promoting M2 macrophage polarization for immunomodulation [38]. The drug TH was effectively loaded onto the PDA NPs through π–π stacking interactions, facilitating its subsequent, on-demand release upon NIR irradiation. Therefore, the hydrogel system (CS-MCP/PVA/PDA@TH) can achieve synergistic antibacterial and anti-inflammatory effects in the early stage of infection. During the cell proliferation stage, the hydrogel’s specific ‘CP–PC’ interaction can facilitate endothelial cell adhesion and proliferation and promote angiogenesis. Through investigation using *in vivo* skin wound infection model, the pH/NIR-responsive CS-MCP/PVA/PDA@TH hydrogel demonstrates programmed antibacterial, anti-inflammatory, immunoregulatory and angiogenesis functions, giving it great potential as an effective wound dressing material.

## Materials and methods

### Materials

CS (MW = 200 kDa, AR, 90% deacetylated), PVA (AR, alcoholysis degree = 98–99% (mol/mol), viscosity = 54–66 mPa·s) were obtained from Shanghai Aladdin Biochemical Technology Co., Ltd. (Shanghai, China). TH (98%, AR) and dopamine hydrochloride (DA, 98%, AR) were obtained from Macklin Reagents Co., Ltd. (Shanghai, China). The antibodies of type I collagen, type III collagen, tumor necrosis factor-α (TNF-α), CD31, CD86 and CD206 were obtained from Affinity Biosciences Co., Ltd. (Cincinnati, USA).

### Preparation and characterizations of PDA NPs

Synthesis of PDA NPs using one-pot method [39, 40]. One gram of DA was added into mixed solvent of aqueous ammonia, ethanol and deionized (DI) water (1.5/80/180 mL) under stirring at 25°C for 24 h. The solution of PDA NPs was collected by centrifugation, and subsequently dried under vacuum. The PDA NPs were characterized by scanning electron microscopy (SEM; QUANTA200, USA), dynamic light scattering (DLS; Nano-ZS90, Malvern Panalytical, Malvern, UK), Fourier-transform infrared (FTIR) spectrometry (Nicolet iS50, Thermo Scientific, USA) and X-ray photoelectron spectrometer (XPS, K-Alpha, Thermo Scientific, USA).

### Loading efficiency of PDA NPs with TH

Ten milligrams of PDA NPs were suspended in 20 mL of TH solution and incubated for 24 h. The mixture TH-loaded PDA NPs (PDA@TH) was subsequently collected via centrifugation, rinsed three times with DI water and freeze-dried. The adsorption properties of the PDA NPs were measured by UV–visible spectrophotometry (UV-3600; Shimadzu, Kyoto, Japan).


(1) TH loading ratio (%) = mass of TH loaded on PDA NPs/(mass of TH loaded on PDA NPs + mass of PDA NPs).

### Preparation of hydrogels

About 2 wt% of CS-MCP was added into different contents of PVA (4 wt%, 6 wt% and 8 wt%) under constant agitation for 3 h at 90°C to form the solutions (1:2, 1:3 and 1:4). The stable hydrogels (CS-MCP/PVA_1:2_, CS-MCP/PVA_1:3_ and CS-MCP/PVA_1:4_) were formed by three times of freeze–thaw cycle. The conditions were set at −20°C for freezing and 25°C for thawing, with 12 h freezing and 4 h thawing. About 0.4 wt% of PDA@TH was dispersed into the CS-MCP/PVA_1:3_, and then using three times of freeze–thaw cycles to form CS-MCP/PVA/PDA@TH hydrogel. The content of groups are shown in Table S1.

### Characterization of hydrogels

The morphological structures of the hydrogels were examined using SEM. The swelling ratio (SR) of hydrogels in PBS after swelling for 48 h was calculated using the following formula:


(2) $$\mathrm{SR}\;(\%)=\frac{W_{w}-W_{d}}{W_{d}}\times \;100\%,$$

where *W_w_* as wet weight, *W_d_* as dry weight. The test was repeated thrice.

The viscoelastic properties of the hydrogels were analyzed through dynamic rheological properties at room temperature. The mechanical properties and rheological properties of the hydrogels were characterized by rheometer (DISCOVERY HR-1 TA) and universal mechanical testing machine (INSTRON 5575), respectively. All measurements were repeated thrice.

Fresh porcine skin was cleaned and dried with PBS. Samples with a 2 cm^2^ adhesive area were prepared and tested using standard overlapping shear tests and mechanical testing machines by sandwiching the hydrogel between two pieces of pig skin at 10 mm/min. The maximum tensile force and maximum shear force were extracted from the force–displacement curve as key quantitative indicators for evaluating the hydrogel’s adhesion performance. A plastic plate was used as the rigid substrate for both the tissue and hydrogel. All measurements were repeated thrice.

The antioxidant activity of CS-MCP/PVA/PDA@TH was evaluated via the DPPH radical scavenging assay. Briefly, a volume-equivalent amount of DPPH ethanol solution (0.1 mM) was combined with the hydrogel extract followed by incubation in darkness for 30 min and subsequent wavelength scanning via a UV-visible spectrophotometer. The DPPH degradation rate was calculated using Equation (3),


(3) $$\mathrm{DPPH}\;\mathrm{clearance}\;\mathrm{rate}\;(\%)\;=\;1-\;(H_{B}-H_{S})/H_{c}\;\times \;100\%,$$

where *H*_B_ as blank control (DPPH + ethanol), *H*_C_ as negative control (DPPH + RO) and *H*_S_ as samples (DPPH + hydrogel extract). All measurements were repeated thrice.

### Photothermal effect of PDA NPs and CS-MCP/PVA/PDA hydrogel

Infrared thermal imaging camera (Testo 885-2, Germany) was used to record the temperature and image of PDA NPs and CS-MCP/PVA/PDA hydrogel in real-time. PDA and PDA@TH NPs were irradiated with at 808 nm laser (1.0 W/cm^2^) for 10 min. CS-MCP/PVA/PDA hydrogel was irradiated (0.1, 0.5 or 1 W/cm^2^) for 10 min. Hydrogel was irradiated (0.5 W/cm^2^, 600 s) and followed by natural cooling for four cycles; the temperature changes during the entire process were recorded. The photothermal conversion efficiency (*η*) of hydrogel was calculated according to Equations (4)–(7)


(4) $$\eta \;=\;hA(T_{\max,\;\mathrm{sam}}-T_{\mathrm{sam},\;\mathrm{water}})/I\;(1-10^{A-808}),$$

(5) $$hA\;=\;m_{D}C_{D}/\tau_{s},$$

(6) $$t\;=\;-\tau_{s}\;\ln\;\theta ,$$

(7) $$\theta \;=\;T(t)-T_{\mathrm{sur}}/T_{\max,\mathrm{sur}}-T_{\mathrm{sur}},$$

where *T*_max,sam_ represents the maximum temperature induced by samples; *T*_max,water_ represents the maximum temperature induced by water; *I* represents the laser power; A-808 represents the absorbance of samples aqueous solution at 808 nm; *m*_D_ represents the weight of water; *C*_D_ represents the heat capacity of water; *t* is the time during the cooling period and *τ_s_* is the time constant for heat transfer of the system.

### Intelligent response to drug release

The intelligent drug release behavior of CS-MCP/PVA/PDA@TH hydrogel was studied under two different conditions, i.e. NIR and an acidic environment. First, 0.5 g of CS-MCP/PVA/PDA@TH hydrogel was immersed in PBS (5 mL, pH = 7.4) and then irradiated (1.0 W/cm^2^, 10 min). The optical density (OD) value of TH was measured by a UV–visible spectrophotometer (thrice for verification). Next the *in vitro* release behavior of TH was investigated by shaking (100 rpm) at 37°C, same as the above method under pH conditions of 5.0 and 7.4 at different times. The cumulative drug release formula:


(8) $$\mathrm{Cumulative}\;\mathrm{release}\;(\mathrm{mass})\;=\;5C_{n}\;+\;\Sigma C_{n-1},$$

where *C_n_* and *C_n_*_−1_ were the concentrations of TH in the solutions collected for *n* and *n* − 1 (*n* ≥ 1) times, respectively.

### Antibacterial activity evaluation

*E. coli* (ATCC 25922) and *S.aureus* (ATCC 6538) were used to evaluate the antibacterial activity of the sterilized hydrogels (three parallel samples). One milliliter of *S.aureus* suspension (1 × 10^5^ CFU/mL) was added to the hydrogel and incubated at 37°C, 24 h. The OD values of the suspension were measured using a microplate reader at 600 nm. Additionally, 100 μL bacterial suspension was plated on agar plates, incubated at 37°C for 24 h under humid conditions, with colony counts performed afterward and a hydrogel-free control group included for comparison. Next, hydrogels (∅5 mm) were onto the *S.aureus*-coated agar plate and incubated at 37°C. After incubation for 24 h, the diameters of the inhibition zones were measured by the Oxford cup method [41]. The morphology of *S.aureus* in the control and CS-MCP/PVA/PDA@TH + NIR groups were fixed with 2.5% glutaraldehyde, rinsed with PBS, dehydrated with ethanol. After freeze-drying, the samples were sprayed with gold, and the morphology of bacteria was observed under SEM. In particular, the group of CS-MCP/PVA/PDA@TH + NIR was irradiated (1.0 W/cm^2^, 10 min) and then incubated. All of tests of *E.coli* were carried out following the same procedures.

### Cytocompatibility evaluation of the hydrogels

To evaluate the cytotoxicity of the hydrogels, human umbilical vein endothelial cells (HUVECs) were directly cultured with the hydrogels. HUVECs were seeded into 48-well plates (5 × 10^3^ cells/well) and incubated for 4 h before sterilized hydrogel disks were placed into the wells. After incubation for 24 h, the cell survival ability of HUVECs was evaluated by CCK-8 kit and Live/Dead staining kit; the cytoskeleton morphology was stained with rhodamine-phalloidin and DAPI, and following observed with a fluorescence inverted microscope. Following incubation for 3 days, cell proliferation and viability were evaluated under hydrogels using the CCK-8 assay. Cell viability formula:


(9) $$\mathrm{Cell}\;\mathrm{viability}\;(\%)\;=\;\frac{{OD}_{C}-{OD}_{B}}{{OD}_{A}-{OD}_{B}}\times 100\%,$$

where *OD_A_* as negative control, *OD_B_* as blank control and *OD_C_* as experimental group.

### Tube formation assay of the hydrogels

HUVECs were seeded in 48-well plates precoated with Matrigel (10 mg/mL), after which 0.25 mL of the respective group solution was added to each well, and the plates were incubated at 37°C with 5% CO_2_. After incubation, the morphology of the HUVECs was observed using an inverted microscope, and digital images were captured for quantitative analysis of branch point number and total tube length. The extraction method entailed immersing UV-sterilized hydrogels (prepared at the same concentration as the CS-MCP solution) in cell culture medium within centrifuge tubes at 4°C for 12 h.

### Cell migration assay

HUVECs were plated in 6-well plates (density of 3 × 10^5^ cells/mL) and incubated 12 h. A pipette tip (200 µL) was employed to gently scratch the cell monolayer to create a wound mimic, and were rinsed three times with PBS to clarify the scratch area. Then, the cells were treated with different hydrogels. Following an additional 24 h incubation period, cell migration was visualized using an optical microscope, and the migrated area was quantified *via* Image J software.

### M2 polarization of macrophages

The different groups (CS/PVA, CS-MCP/PVA, CS-MCP/PVA-PDA and PDA NPs) treated the RAW 264.7 macrophages with LPS (200 ng/mL) for 24 h. After treatment, RAW 264.7 macrophages were fixed with 4% paraformaldehyde for 30 min, permeabilized with 0.1% Triton X-100 for 40 min, blocked with 2% BSA for 1 h and then labeled with primary antibodies against CD86 and CD206 (at 2 µg/mL) overnight at 4°C. The secondary antibody (4 µg/ml) was incubated for 1 h at 25°C, and the DAPI stained for detection using a fluorescence microscope.

### Transcriptome analysis of HUVECs

As for RNA sequencing assay, HUVECs were seeded in 6-well plates (density of 4 × 10^5^ cells/well). Following 3 days of incubation, HUVECs from each group were harvested, and total RNA was extracted and purified using Trizol reagent (Sigma, USA). The RNA libraries were sequenced by OE Biotech, Inc., Shanghai, China. Functional enrichment analysis (*P* values <0.05) including Kyoto Encyclopedia of Genes and Genomes (KEGG) pathways and genome enrichment analysis were carried out to analyze the potential biological mechanism.

### *In vivo* experiments of wound healing

The animal experiments were conducted following the protocols adopted by the Medical Ethics Committee of Southwest Jiaotong University (SWJTU-2103-023) and followed the guidelines of laboratory animal administration rules of China. Sprague–Dawley (SD) rats (male; 8 weeks old; 180–220 g;) were anesthetized with gas anesthesia, followed by dorsal depilation (shaving) and punching perforation to create full-thickness skin defects (10 mm diameter) on their dorsal regions. The wound sites were infected with 10 μL of *S.aureus* suspension (1 × 10^7^ CFU/mL). After infection, rats were treated with six groups of CS/PVA, CS-MCP/PVA, CS-MCP/PVA/PDA + NIR, CS-MCP/PVA/PDA@TH, CS-MCP/PVA/PDA@TH + NIR (1.0 W/cm^2^, 10 min) and normal saline as the control group. During the treatment, *in vivo* thermal imaging was performed to capture real-time infrared images and corresponding temperature data using a thermal imaging camera. The wound coalescence area (%) formula:


(10) $$\mathrm{Wound}\;\mathrm{coalescence}\;\mathrm{area}\;(\%)\;=\;\frac{C_{0}-C_{t}}{C_{0}}\times 100\%,$$

where *C*_0_ as the initial wound area at day 0, *C_t_* as the wound area healing at time *t*, day 3, 7 or 14.

The *in vivo* antibacterial efficacy against *S.aureus* was evaluated on day 1 post-treatment.

### Histologic analysis

The skin tissues from the wound regions were collected at day 0, 3, 7 and 14 after treatment, and then stained with hematoxylin and eosin (H&E) as well as Masson’s trichrome staining, and observed under a microscope.

### Immunohistochemical analysis

To perform immunohistochemical staining, the slides were fixed and frozen in 5% BSA solution, then incubated overnight at 4°C with rabbit polyclonal primary antibodies (anti type I collagen, anti-type III collagen, anti TNF-α, anti CD31, anti CD86 and anti CD206), followed by secondary antibody incubation at 4°C for 2 h. Slides were observed and photographed using a microscope.

### Statistical analysis

All data were presented as the mean ± standard deviation (SD). Statistical differences were identified using one-way analysis of variance (ANOVA), followed by Tukey’s *post hoc* test for pairwise comparisons. Each experiment was independently conducted at least three times to guarantee experimental reproducibility. Statistical significance was defined as *P < 0.05, **P < 0.01, ***P < 0.001, ****P < 0.0001.

## Results and discussion

### Synthesis and characterization of PDA NPs and PDA@TH NPs

**Figure 2:**
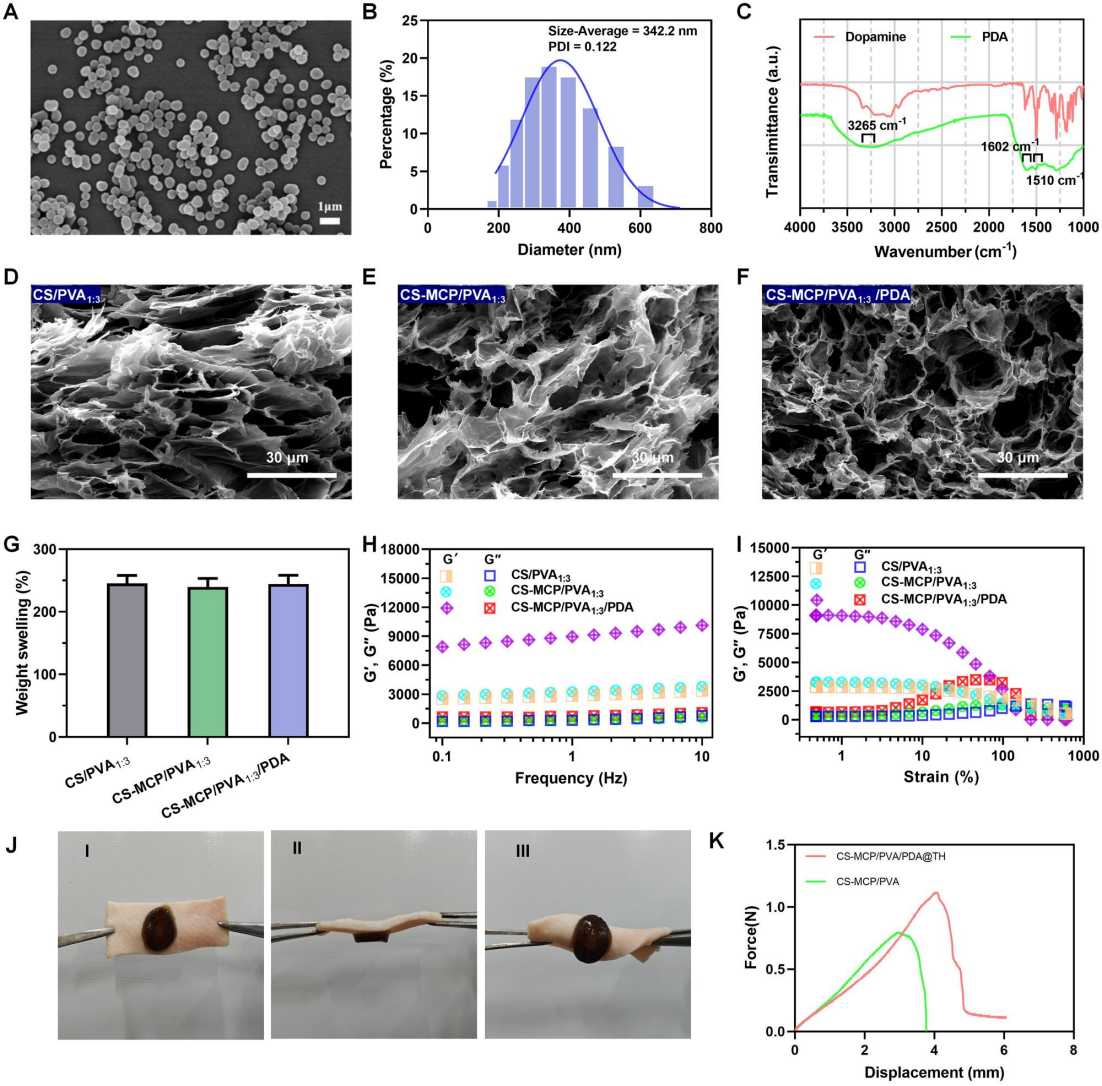
(**A**) SEM morphology of PDA NPs. (**B**) Particle size distribution of PDA NPs. (**C**) FTIR spectrometry of dopamine and PDA. (**D–F**) Structural and morphological characterization of CS/PVA_1:3_, CS-MCP/PVA_1:3_ and CS-MCP/PVA_1:3_/PDA hydrogels. (**G**) SR of hydrogels (data are means ± SD, *n* = 3). (**H, I**) Rheological properties of hydrogels with changing frequency and strain. (**J**) Adhesion images of CS-MCP/PVA/PDA@TH hydrogel on pig skin: (I) vertical, (Ⅱ) inverted, (III) folded. (**K**) The adhesion curves of different hydrogels were obtained.

To prepare NIR photothermal responsive hydrogels, PDA NPs were first synthesized. PDA NPs exhibited a spherical structure with a mean particle diameter of 342.2 nm (Figure 2A and B). FTIR spectroscopy of PDA showed characteristic absorption bands at 1510 and 1602 cm^−1^, attributed to the N–H vibrational modes within aromatic ring moieties, and the absorption signal at 3265 cm^−1^ indicated the presence of –OH groups derived from 1,2-dihydroxybenzene or catechol (Figure 2C). Subsequently, a drug loading efficiency of PDA@TH was 9.2% (Figure S1, Supporting Information). Furthermore, in the XPS spectra of PDA@TH NPs, the detection of Cl 2p signals in PDA@TH indicates successful loading of TH. The relative areas of C=O and O–C=O components in the C 1s spectrum of PDA@TH show a significant increase, with a corresponding rise in the proportion of C=O components in the O 1s spectrum (Figure S2, Supporting Information).

### Characterizations of hydrogels

All groups of hydrogels were formed by repeated freezing and thawing and cross-linked by hydrogen bonds to form a stable network structure without any chemical crosslinking agent. The SEM images show that the hydrogels presented a macroporous, sponge-like structure and the pores of the porous structure become smaller with the increase in PVA content (Figure 2D and F, Figure S3, Supporting Information). The internal pores of CS-MCP_1:3_/PVA/PDA hydrogel were more uniform and compact, attributed to the cross-linking between PDA NPs and the CS-MCP/PVA hydrogel, while tensile and compression tests further confirmed that the incorporation of PDA NPs enhanced the hydrogel’s mechanical strength (Figure S4, Supporting Information).

Owing to the increase in PVA content, the SRs of CS-MCP/PVA hydrogels at mass ratios of 1:2, 1:3 and 1:4 reached 1.75, 2.5 and 2.75 times their original volumes, respectively (Figure 2G and Figure S5, Supporting Information). The swell ratios of the CS-MCP/PVA_1:3_ and CS-MCP_1:3_/PVA/PDA hydrogels were similar to that of the CS/PVA_1:3_ hydrogel (Figure 2G). Collectively, these favorable water absorption and retention properties enable the hydrogels to efficiently absorb wound exudate and thereby accelerate the wound healing process [41].

Dynamic oscillatory frequency sweep tests of the hydrogels were conducted, all hydrogels exhibited gel-like elastic behavior (*G*′ ≫ *G*″) across the tested frequency range (Figure 2H and Figure S6A, Supporting Information). Notably, CS-MCP/PVA_1:3_/PDA hydrogel displayed higher *G*′ and *G*″ values than other formulations, reflecting enhanced elasticity and elevated energy storage modulus attributed to PDA NPs. This was associated with PDA-mediated cross-linking with the CS-MCP/PVA network, which restricted chain mobility and strengthened the hydrogel’s elastic response. Furthermore, strain amplitude sweep tests revealed a *G*′–*G*″ crossover at a critical strain for all hydrogels, indicating network disruption at this threshold (Figure 2I, Figure S6B, Supporting Information); beyond this strain, *G*′ < *G*″ was observed, confirming progressive network collapse with increasing strain. Given its typical rheological performance, the CS-MCP/PVA1:3 hydrogel was selected as the model for subsequent investigations.

To assess the adhesive performance of the CS-MCP/PVA/PDA@TH hydrogel, tensile and mechanical adhesion tests were conducted on porcine skin. Specifically, the hydrogels maintained robust adhesion to the porcine skin surface even after undergoing mechanical manipulations such as vertical suspension, flipping and folding (Figure 2J), and under identical experimental conditions, they exhibited significantly higher adhesive strength than the CS-MCP/PVA hydrogel (Figure 2K). These excellent adhesive properties are primarily attributed to the adhesive effect of PDA NPs. In addition to these superior performances, the hydrogel also demonstrates remarkable ROS scavenging capacity—synergistically mediated by PDA NPs, with a ROS clearance rate of approximately 99% within 30 min (Figure S7, Supporting Information).

### Photothermal effect of CS-MCP/PVA/PDA hydrogel and PDA@TH NPs

**Figure 3:**
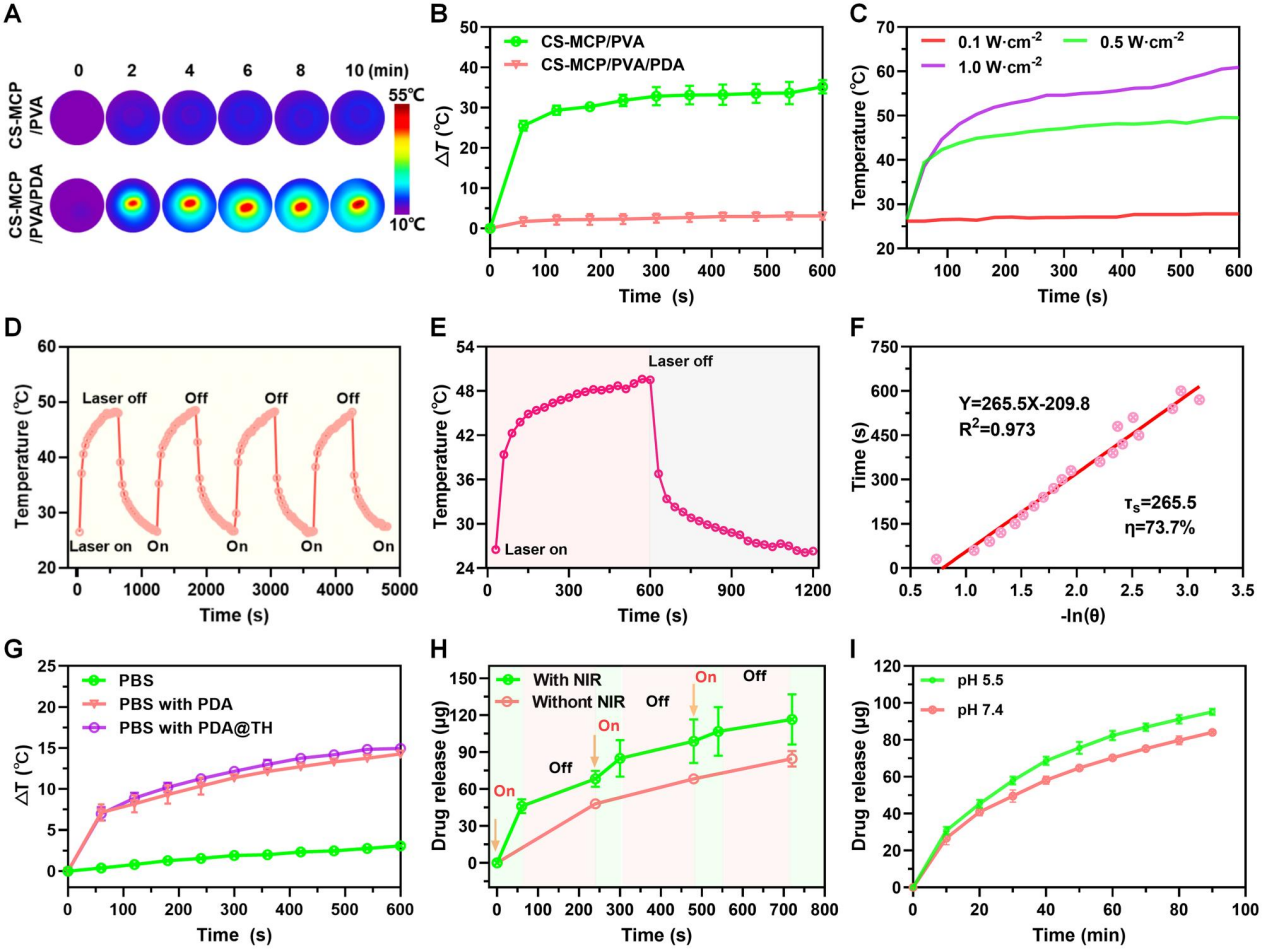
(**A**) Infrared imaging of hydrogels with or without PDA NPs. (**B**) Temperature increase curve of hydrogels with and without PDA NPs. (**C**) Temperature change curves of CS-MCP/PVA/PDA hydrogel during NIR laser irradiation at different powers. (**D**) Photothermal curves of CS-MCP/PVA/PDA hydrogel for four heating/cooling cycles. (**E**) ‘On—off’ temperature change of CS-MCP/PVA/PDA hydrogel (0.5 W/cm^2^). (**F**) The corresponding linear relationship between −ln(θ) and time (s). (**G**) Temperature changes of PDA and PDA@TH NPs (1.0 W/cm^2^). (**H**) NIR-responsive drug release curve of TH-loaded hydrogels (‘on’ indicates irradiation, ‘off’ indicates no irradiation). (**I**) pH-responsive drug release curve of CS-MCP/PVA/PDA@TH hydrogel.

As previously reported, PDA NPs have high efficiency in converting NIR laser irradiation into heat [42]. Figure 3A shows that the CS-MCP/PVA/PDA hydrogels continued to heat up and reached 49.6°C (maximum temperature), whereas the CS-MCP/PVA hydrogel did not exhibit any NIR-responsive photothermal behavior. Figure 3B provides the temperature rise trend of the hydrogels, and the CS-MCP/PVA/PDA hydrogel shows a temperature rise difference of approximately 35°C. With increasing power densities, groups’ temperature increased to 27.8°C, 49.6°C and 60.9°C, respectively (Figure 3C).

Figure 3D shows that all of the groups were near identical, confirming the photothermal stability and the heat resistance of the hydrogel. Notably, CS-MCP/PVA/PDA hydrogel has good photothermal conversion efficiency (Figure 3E and F, *η* = 73.7%). Figure 3G shows that TH loading on the PDA NPs has no obvious effect on the photothermal effect of PDA NPs; the temperature gradually increased from 18 to 40°C. The remarkable photothermal conversion ability of CS-MCP/PVA/PDA hydrogel can facilitate controlled drug release and hold promise as a highly effective NIR-responsive photothermal therapeutic agent.

### Intelligent pH/NIR-responsive drug release

The drug release behaviors of CS-MCP/PVA/PDA hydrogel under NIR and different pH values were investigated. Initially, the TH release behavior was measured. Figure 3H shows that TH release was considerably enhanced under NIR irradiation from the CS-MCP/PVA/PDA@TH hydrogel under a NIR-triggered on-off mechanism. At pH = 5.5, the release rate of TH from the CS-MCP/PVA/PDA@TH hydrogel was higher (Figure 3I). CS comprises a considerable number of amino groups, which can be protonated in an acidic environment to exhibit pH-responsive behavior. CS-MCP remains rich in amino, hydroxyl and carboxyl groups, and pH-responsive drug release behavior of hydrogel is attributed to electrostatic repulsion arising from the protonated amine groups of PDA and cationic TH molecules.

### Antibacterial activity

**Figure 4:**
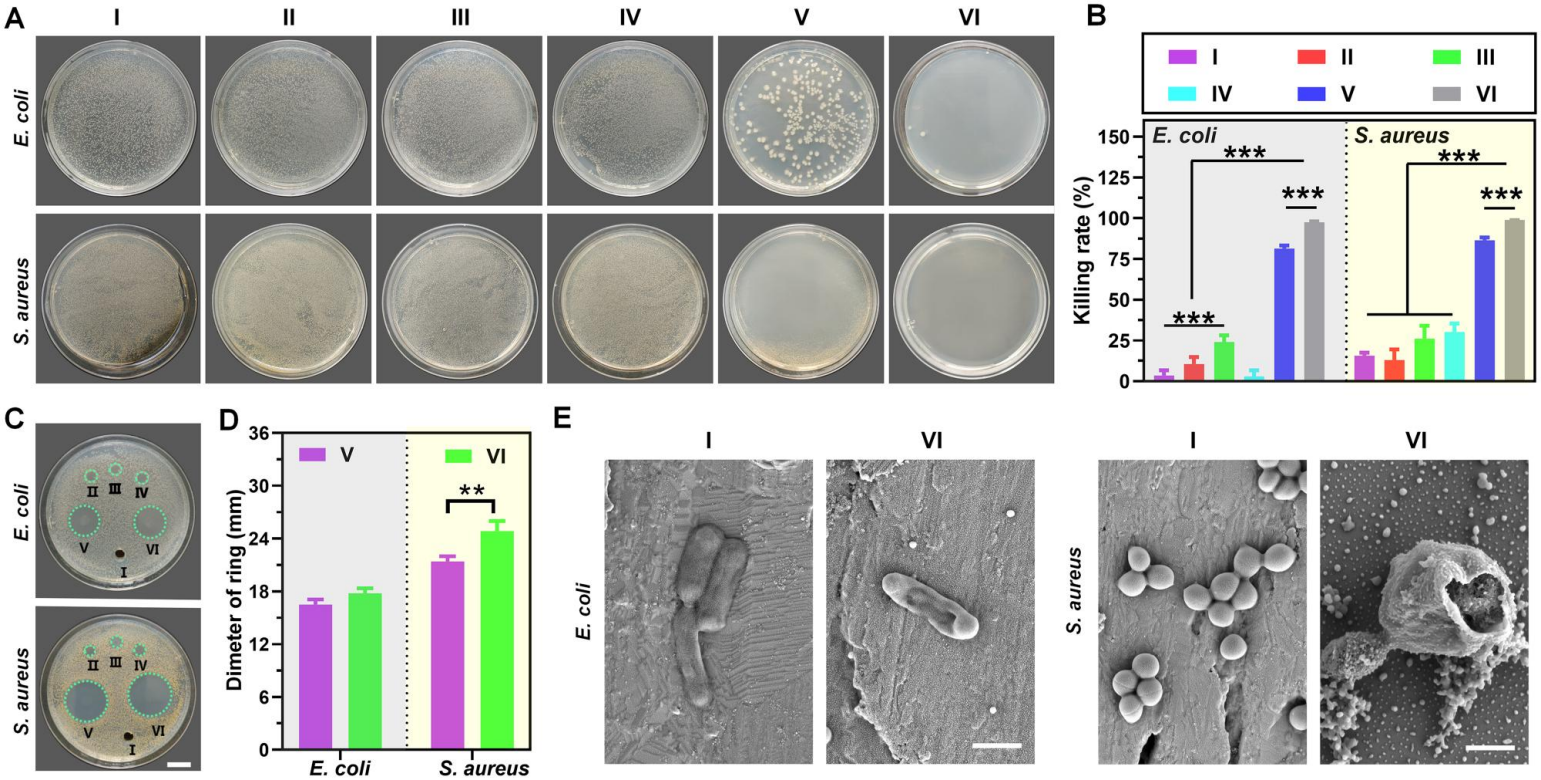
Antibacterial activity (I: control; Ⅱ: CS/PVA hydrogel; III: CS-MCP/PVA hydrogel; IV: CS-MCP/PVA/PDA hydrogel; Ⅴ: CS-MCP/PVA/PDA@TH hydrogel; Ⅵ: CS-MCP/PVA/PDA@TH hydrogel + NIR). (**A**) Bacterial growth on the coating plate after the co-culture of hydrogel and bacteria. (**B**) The killing rate of *E.coli* and *S.aureus* (*n* = 6). (**C**) Photos of hydrogels producing inhibitory zones against *E.coli* and *S.aureus*. (**D**) The size of the bacteriostatic zone formed by different hydrogels (*n* = 3). (**E**) SEM images of bacterial morphology. Scale bar: 500 nm.

The ideal wound dressing, besides acting as a physical barrier to extrinsic bacterial colonization, should also have intrinsic antibacterial activity [39, 40]. After co-culture of each group of materials with bacteria, the groups of CS-MCP/PVA/PDA@TH hydrogel (group Ⅴ) and CS-MCP/PVA/PDA@TH hydrogel + NIR (group Ⅵ) both exhibited good antibacterial activity (Figure 4), primarily attributed to the inherent antibacterial properties of TH itself. Notably, NIR irradiation promoted the accelerated release of TH, thereby endowing group Ⅵ with excellent antibacterial efficacy (Figure 4A). The antibacterial rate of group Ⅴ was more than 80%, and the antibacterial rate of group Ⅵ could achieve more than 97% (Figure 4B). The inhibition zone of group Ⅴ was 13 mm (*E.coli*) and 19 mm (*S.aureus*), respectively. Moreover, in group Ⅵ, with the NIR introduction, the antibacterial effect was significantly enhanced, with the inhibition zone of 16 mm for *E.coli* and 26 mm for *S.aureus* (Figure 4C and D). In addition, all groups exhibited moderately stronger antibacterial activity against *S.aureus* relative to *E.coli*, attributed to the outer membrane of Gram-negative bacteria conferring greater resistance to bactericidal and bacteriostatic agents [43]. In addition, Figure 4E displays the morphology of bacteria treated with group Ⅵ, revealing that the membranes of both bacterial strains exhibited varying degrees of collapse and damage upon exposure to TH and NIR irradiation. These results indicated that the excellent bactericidal effect of CS-MCP/PVA/PDA@TH hydrogel in this study was due to the combination of NIR and TH.

### Biological functionality evaluation

**Figure 5:**
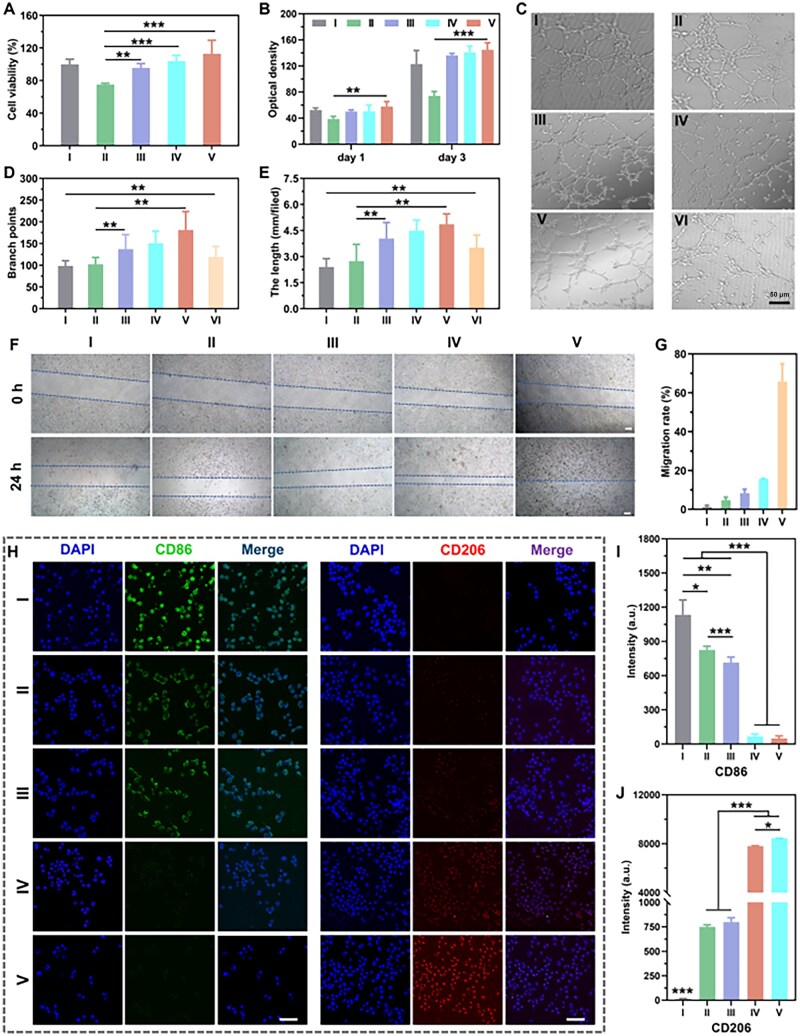
Cytocompatibility, angiogenesis of HUVECs and immunoregulatory activity of RAW 264.7 cells. (**A**) Cytocompatibility evaluation of different hydrogels (I: Control; II: CS/PVA hydrogel; III: CS-MCP/PVA hydrogel; IV CS-MCP/PVA/PDA hydrogel; V: CS-MCP/PVA/PDA@TH hydrogel; VI: CS-MCP solution). (**B**) HUVECs proliferation. (**C**) Tube formation images of different hydrogels. (**D**) Tube branch points treated with different hydrogels. (**E**) Tube length treated with different hydrogels. Image of (**F**) scratches micrographs and (**G**) migration rate. (**H**) Immunofluorescence staining images of hydrogels (I: Control; II: CS/PVA hydrogel; III: CS-MCP/PVA hydrogel; IV: CS-MCP/PVA/PDA hydrogel; V: PDA NPs). (**I**) Fluorescence intensity of CD86. (**J**) Fluorescence intensity of CD206. (*n* = 5). Scale bars: 50 μm. (*n* = 5, *P < 0.05, **P < 0.01, ***P < 0.001).

Biocompatibility and angiogenesis activity of the hydrogels are shown in Figure 5. Compared to the CS/PVA hydrogel (group Ⅱ), the CS-MCP/PVA hydrogels (group III) showed better cytocompatibility and cell proliferation properties (Figure 5A and B). This is attributable to the superior biocompatibility of CS-MCP over CS, a property conferred by the CP groups that enhance cell adhesion and proliferation, as reported in our previous work. Live/dead cell staining images (Figure S8, Supporting Information) showed that more live cells were observed in group III and CS-MCP/PVA/PDA (group IV) groups compared to group Ⅱ. The HUVECs presented a well-spread shape with obvious cellular pseudopodia of all groups, indicating that group IV was favorable for HUVECs attachment and proliferation, demonstrating good biocompatibility.

Angiogenesis is essential for wound healing as it facilitates the delivery of nutrients. Compared with the CS/PVA (Ⅱ), the CS-MCP/PVA (III), CS-MCP/PVA/PDA (IV) and CS-MCP/PVA/PDA@TH (Ⅴ) groups showed higher formations of tube-like capillaries (Figure 5C). We also observed that the HUVECs treated with CS-MCP solution (group Ⅵ) spontaneously organized into cords and formed a honeycomb-like network. The tube length and branch points were also assessed as angiogenic parameters, and groups III–Ⅵ had more tubes and branch points (Figure 5D and E). Significant differences in tube length and branch points were found between group III and group Ⅱ (*P *< 0.01). This was attributed to the CP group in CS-MCP, which can facilitate cell adhesion and promote tube formation, as demonstrated in our previous work. We also observed the tube length and branch points in group Ⅴ was highest. In general, CS-MCP and TH in the CS-MCP/PVA/PDA@TH hydrogel promoted the tube formation of HUVECs at the same time, indicating the potential of the CS-MCP/PVA/PDA@TH hydrogel for promoting angiogenesis in skin wound repair.

Evaluating the ability of cells to migrate toward the injury or specific region is crucial for wound healing. As shown in Figure 5F and G, compared with the control group, all the hydrogel groups promoted HUVECs migration, and the CS-MCP/PVA/PDA@TH (group Ⅴ) most significantly accelerated wound healing, with a mobility of 65.7% at 24 h. The results showed that CS-MCP/PVA/PDA@TH had good cytocompatibility and could significantly promote cell proliferation and migration *in vitro*.

### M2 phenotype polarization of macrophages

Macrophage polarization serves a pivotal role in wound repair processes. M1 macrophages release pro-inflammatory mediators, whereas M2 macrophages secrete anti-inflammatory cytokines and reparative growth factors—effectively mitigating the inflammatory cascade and promoting wound contraction [44]. Immunofluorescence staining (Figure 5H) confirmed the polarization of macrophages after LPS stimulation. RAW 264.7 cells underwent a phenotypic shift toward the M1 subtype, characterized by CD86-positive and CD206-negative expression. Both PDA NPs (group Ⅴ) and CS-MCP/PVA/PDA (group IV) exhibited CD86 negativity and CD206 positivity. For these two experimental groups, CD86 fluorescence intensity was markedly reduced compared to the other groups, whereas CD206 fluorescence intensity was significantly elevated (Figure 5I and J). Collectively, the combination of PDA NPs and CS-MCP/PVA hydrogel exhibits distinct immunomodulatory activity.

### RNA sequencing analysis

**Figure 6:**
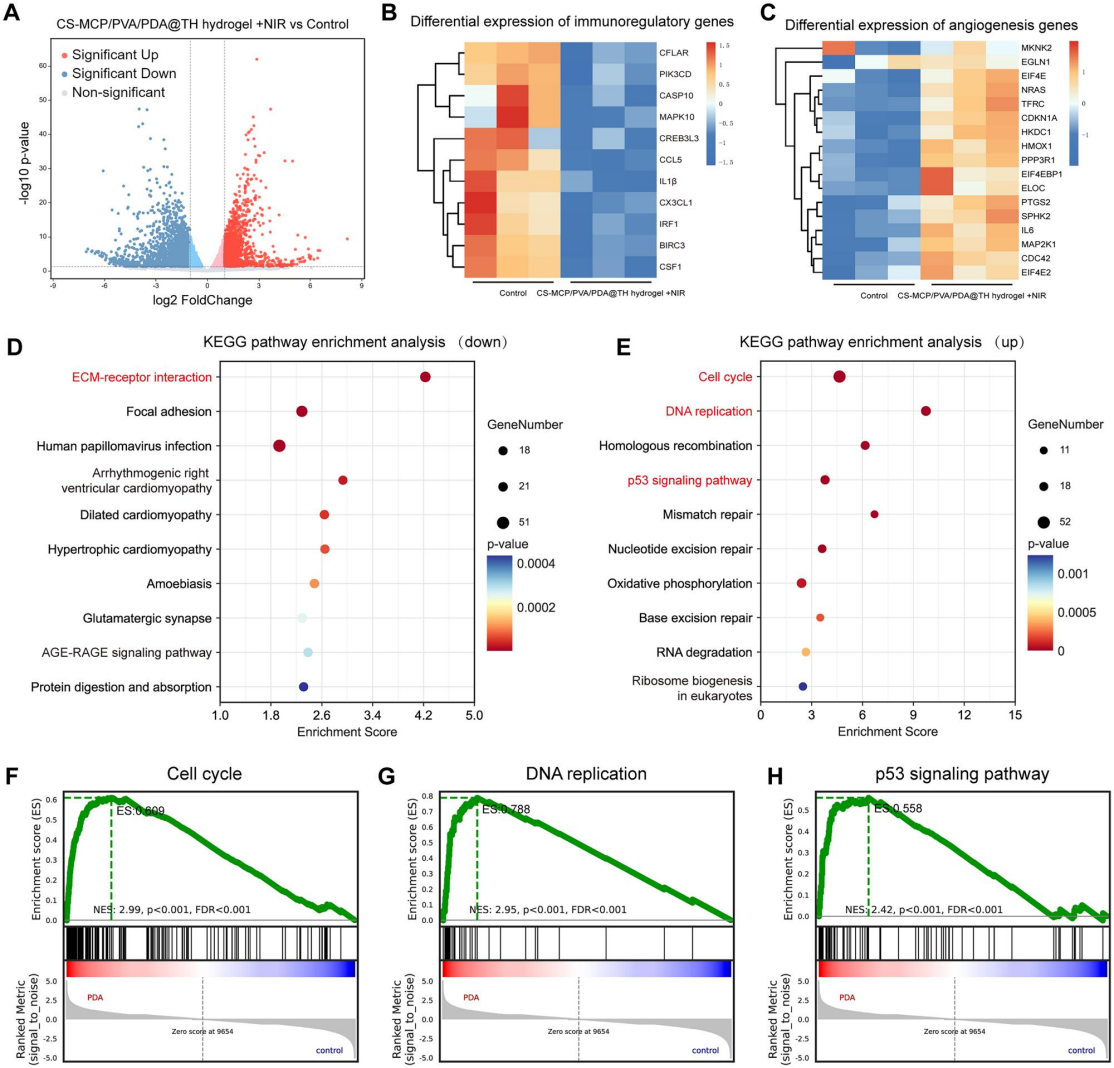
RNA sequencing analysis. (**A**) Volcano plot (|log_2_FoldChange| > 1, *P *< 0.05). (**B, C**) Differential gene expression clustering heatmap. (**D, E**) Down/upregulated differential gene KEGG enrichment analysis. (**F–H**) GSEA enrichment analysis.

To exploit the molecular mechanism involved in wound healing based on hydrogel dressings, RNA-seq analysis was performed on the control group and the CS-MCP/PVA/PDA hydrogel + NIR group. Before performing RNA-seq analysis, we differentially treated HUVECs for 3 days. A total of 3235 differentially expressed genes (DEGs) were detected between CS-MCP/PVA/PDA hydrogel + NIR group and the control group (|log_2_FC| > 1.0, *P *< 0.05), volcano plots revealing 1452 upregulated and 1783 downregulated genes (Figure 6A). Further focusing on the DGEs between the CS-MCP/PVA/PDA hydrogel + NIR group and the control group, we found that the treatment significantly suppressed the expression of inflammation-related factors while promoting the upregulation of angiogenesis-related factors (Figure 6B and C). To gain a deeper understanding of the biological mechanisms behind these changes in gene expression, we performed enrichment analysis using the KEGG. The results showed that the downregulated genes were mainly enriched in signaling pathways such as extracellular matrix–receptor interactions, which may contribute to attenuating the inflammatory response and fibrotic process at the wound site. On the contrary, the upregulated genes were significantly enriched in key signaling pathways such as cell cycle regulation, DNA replication and cell proliferation (Figure 6D and E), which directly supported the idea that hydrogel combined with NIR treatment could enhance cell proliferation. In addition, the above findings were further consolidated by the results of GSEA, which confirmed the positive regulatory effects of CS-MCP/PVA/PDA hydrogel + NIR treatment on cell cycle, DNA replication and cell proliferation by systematically evaluating the degree of enrichment of the gene set in a specific biological process (Figure 6F–H). The positive effects of this CS-MCP/PVA/PDA hydrogel + NIR group in promoting cell proliferation, enhancing angiogenic function and wound healing were demonstrated.

### *In vivo S.aureus*-infected wound healing

**Figure 7:**
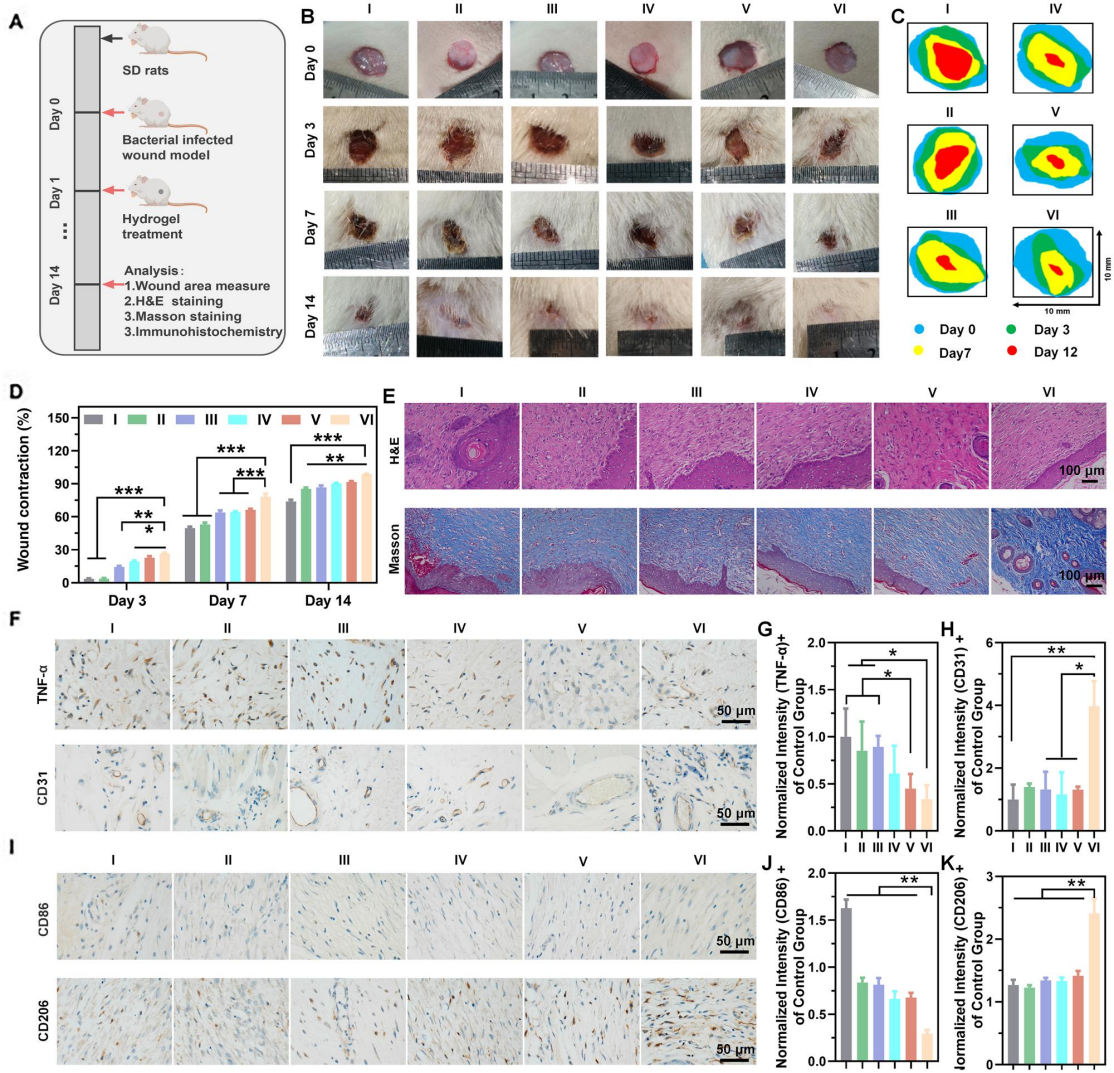
*In Vivo* wound repair (I: control; Ⅱ: CS/PVA hydrogel; III: CS-MCP/PVA hydrogel; IV: CS-MCP/PVA/PDA hydrogel + NIR; Ⅴ: CS-MCP/PVA/PDA@TH hydrogel; Ⅵ: CS-MCP/PVA/PDA@TH hydrogel + NIR). (**A**) Schematic of the *S.aureus*-infected rat skin defect model and hydrogel-based treatment. (**B**) Representative images of *S.aureus*-infected rat skin wounds across treatment groups. (**C**) Wound boundary dynamics during *in vivo* healing; different colored shapes indicate wound areas at each time point. (**D**) Wound contraction analysis of rats in different treatment groups. (**E**) H&E and Masson’s trichrome staining of infected wound tissues 14 days post-treatment. (**F**) Immunohistochemical staining for TNF-α and CD31 expression. (**G**) Quantitative analysis of TNF-α. (**H**) Quantitative analysis of CD31. (**I**) Immunohistochemical staining for CD86 and CD206 expression. (**J**) Quantitative analysis of CD86. (**K**) Quantitative analysis of CD206.

*In vivo*, the programmable antibacterial, anti-inflammatory, immunomodulatory, pro-angiogenic and wound healing-promoting capacities of CS-MCP/PVA/PDA@TH hydrogel under NIR were evaluated using a *S.aureus*-infected rat skin defect model (Figure 7A). Representative wound contraction images and closure rates of each treatment group were documented to assess the healing process (Figure 7B–D). As time progressed, the wound closure rates of all groups increased, and the wound sizes decreased. As we expected, the CS-MCP/PVA/PDA@TH hydrogel + NIR (group Ⅵ) exhibited a smaller wound size at 3 days. Additionally, the number of bacterial colonies in the CS-MCP/PVA/PDA@TH hydrogel + NIR (group Ⅵ) was significantly reduced, and the hydrogel exhibited a strong ability to resist bacterial growth. This indicates that NIR can enhance the antibacterial effect of the CS-MCP/PVA/PDA@TH hydrogel as an auxiliary (Figure S9, Supporting Information). At day 14, the wound closure rate of group Ⅵ was fastest, achieving a high wound contraction ratio of 99% (Figure 7D). These findings demonstrate that the CS-MCP/PVA/PDA@TH hydrogel + NIR exhibited the best wound healing efficacy among all the groups.

### Histological analysis

To further study the wound repair process of different hydrogel treatments, histological and immunological analyses of tissues, including thermal damage, tissue morphology, inflammation factors, vascularization and collagen deposition, were performed.

Thermal imaging of rat wound sites during 10 min of 1.0 W/cm^2^ NIR irradiation (Figure S10) identified CS-MCP/PVA/PDA@TH as the primary heat source without abnormal heat diffusion to healthy skin, and HE staining after three treatment cycles demonstrated that the CS-MCP/PVA/PDA@TH + NIR group achieved accelerated wound healing with well-organized granulation tissue, no pathological/cytological signs of thermal injury (e.g. eschar, blisters, cell necrosis) and no thermal damage compared to normal skin, validating the therapy’s safety.

Many inflammatory cells of H&E staining (Figure 7E, Figure S10, Supporting Information) were observed in the wounds of groups I–III, and group Ⅵ at day 3 after treatments, signifying an aggressive infection driven by *S.aureus.* However, group Ⅴ exhibited fewer inflammatory cells, which was attributed to the antibacterial activity of TH [9]. However, group Ⅵ exhibited diminished inflammatory cell infiltration, attributed to the synergistic bactericidal effect of photothermal therapy and TH release. On day 7, all hydrogel groups showed lower inflammation levels than the control group (Figure S11, supporting information), while group Ⅵ achieved the optimal healing outcome of day 14 (Figure 7E), which was characterized by well-formed epithelial–dermal architecture, intact hair follicles and minimal epidermal gaps. Giemsa staining confirmed the lowest bacterial load in group Ⅵ on day 3 (Figure S12, Supporting Information), which also resulted from this synergistic bactericidal mechanism [45]. In conclusion, under NIR irradiation, the hydrogel CS-MCP/PVA/PDA@TH enhanced anti-inflammatory capacity and facilitated wound healing via synergistic bactericidal effects.

Adequate collagen deposition and remodeling enhance tissue tensile strength and improve therapeutic outcomes [46], which were assessed via Masson’s trichrome staining and immunohistochemical staining for type I/III collagen (Figure 7E, Figures S13 and S14, Supporting Information). Over treatment time, collagen deposition increased across all groups, with group Ⅵ exhibiting enhanced deposition characterized by denser, more mature collagen fibers. As key components of the dermal extracellular matrix, types I and III collagen serve pivotal roles in wound healing—early high-level type III collagen deposition facilitates scarless repair. Immunohistochemical staining was used to assess type I/III collagen levels in wound tissues (Figure S13, Supporting Information). At days 3 and 7, all groups showed higher type III collagen expression than type I, with group Ⅵ displaying the strongest expression. Robust type I collagen expression was observed in all groups of day 14, indicating that types I and III collagen synergistically facilitate late stage wound healing.

### Immunohistochemical staining analysis

TNF-α, as a pro-inflammatory cytokine, is one of the main mediators of host inflammatory response, and the expression of TNF-α was assessed to determine inflammatory levels (Figure 7F and G, Figure S15A and B, Supporting Information). Groups Ⅴ and Ⅵ exhibited weaker TNF-α expression, with group Ⅵ displaying the lowest among all the groups. This was attributed to the synergistic bactericidal effect of CS-MCP/PVA/PDA@TH hydrogel under NIR irradiation, thereby mitigating the inflammatory response.

CD31 modulates multiple key pathways during wound healing, including angiogenesis, epithelial regeneration and collagen synthesis [47–49]. The immunohistochemical staining of CD31 in each group were measured (Figure 7F and H, Figure S15C and D, Supporting Information). As shown in Figure 7F and H, groups III, IV, Ⅴ, and Ⅵ showed strong positive expression of CD31 at 14 days. Among all the groups, group Ⅵ showed the most obvious newborn microvessels, indicating that the hydrogels based on CS-MCP molecular can promote HUVECs adhesion and formation of blood vessels due to the specific ‘CP–PC’ effect [28, 29]. The excellent antibacterial effect of the CS-MCP/PVA/PDA@TH + NIR group can also enhance its angiogenesis.

The immunoregulatory responses of the CS-MCP/PVA/PDA@TH hydrogel under NIR were assessed by studying the immunohistochemical staining of CD86 (M1 phenotype marker) and CD206 (M2 phenotype marker). At day 3 after treatment, all the treated groups showed a positive expression of CD86, which indicated an inflammatory response (Figure S16, Supporting Information). Over time, the CD86 expression became weaker of all the groups compared to the control group. Moreover, group Ⅵ showed almost negative expression of CD86 at day 14, and its CD86^+^ intensity was significantly lower at day 3, 7, and 14 (Figure 7I and J, Figure S16A and B, Supporting Information). Conversely, CD206 expression exhibited an inverse trend relative to CD86 across all groups at day 3, 7 and 14, with group Ⅵ showing the strongest CD206 intensity (Figure 7I and K, Figure S16C and D, Supporting Information). These results demonstrated that the CS-MCP/PVA/PDA@TH hydrogel under NIR had immunoregulatory ability during wound healing, which was due to its programmed antibacterial effect and ability to promote angiogenesis.

## Conclusions

In summary, aiming at the difficulty of infected wound repair, we developed a pH/NIR dual-responsive hydrogel with programmed antibacterial, anti-inflammatory, immunoregulatory and angiogenic capacities to accelerate wound healing. The NIR response of hydrogel has promoted TH release, synergistic enhancement of bactericidal effect, synergistically enhances bactericidal activity, and augments its antibacterial and anti-inflammatory capacities. Based on the specific ‘CP–PC’ binding property of the CS-MCP molecule, the hydrogel promoted HUVECs adhesion and proliferation and the formation of cell tubules *in vitro*, as well as angiogenesis *in vivo*, to facilitate the delivery of nutrients. Further, the strong therapeutic effects of the hydrogel were verified in an *S.aureus*-infected wound model established on rats, wherein its programmed antibacterial, anti-inflammatory, immunomodulatory and angiogenic capacities synergistically promoted wound repair.

## Supplementary Material

rbag022_Supplementary_Data
