# Supplementary material for: Intelligent response hydrogel based on choline phosphorylated chitosan programmed repair of infected wounds
Source: Regen Biomater. 2026 Feb 19;13:rbag022. doi: 10.1093/rb/rbag022 (PMC13175249; doi:10.1093/rb/rbag022)
Supplement: rbag022_Supplementary_Data [file rbag022_supplementary_data.docx]

**Supporting Information**

**Intelligent response hydrogel based on choline phosphorylated chitosan** **programmed repair of infected wounds**

Min Lu^1,†^, Tong Sun^2,†^, Chongxu Tang^1,†^, Xinmei Zhang^1^, Shuyue Hao^5^, Mei Yang^4^, Qiangwei Xin^2^, Zhongqiang Zhu^2^, Mingming Ding^2^, Jie Weng^1^***, Zhiqiang Li^1,3^***, Xingyu Chen^1^***, Jianshu Li^2^

*^1^Institute of Biomedical Engineering, College of Medicine, Southwest Jiaotong University, Chengdu 610031, China*

*^2^College of Polymer Science and Engineering, National Key Laboratory of Advanced Polymer Materials, Sichuan University, Chengdu, 610065, China*

*^3^Department of Orthopedics, The General Hospital of Western Theater Command, Chengdu, College of Medicine, Southwest Jiaotong University, 610083, China.*

*^4^Department of Geriatrics, Hospital of Chengdu University of Traditional Chinese Medicine, Chengdu 610075, China*

*^5^College of Life Sciences and Engineering, Southwest Jiaotong University, Chengdu 610031, China*

**Table S1.** Composition of various hydrogels

| Hydrogel | CS  (wt%) | CS-MCP (wt%) | PVA  (wt%) | PDA NPs (wt%) |
| --- | --- | --- | --- | --- |
| CS/PVA_1:3_ | 2 | 0 | 6 | 0 |
| CS-MCP/PVA_1:2_ | 0 | 2 | 4 | 0 |
| CS-MCP/PVA_1:3_ | 0 | 2 | 6 | 0 |
| CS-MCP/PVA_1:4_ | 0 | 2 | 8 | 0 |
| CS-MCP/PVA_1:3_/PDA | 0 | 2 | 6 | 0.4 |


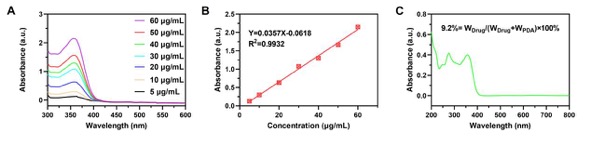


**Figure S1.** (A) Standard curve of TH. (B) Absorbance of different concentrations of TH solutions. (C) Drug-loading of PDA-NPs for TH.


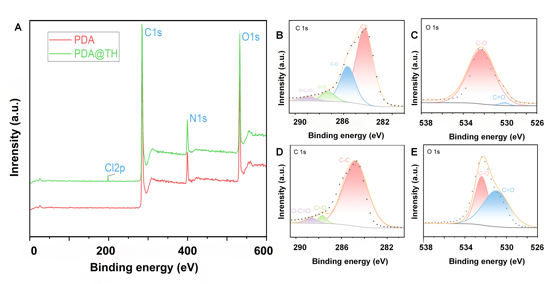


**Figure S2.** (A) The full XPS spectrum analysis of PDA and PDA@TH. (B) C 1s spectrum of PDA. (C) O 1s spectrum of PDA. (D) C 1s spectrum of PDA@TH. (E) O 1s spectrum of PDA@TH.


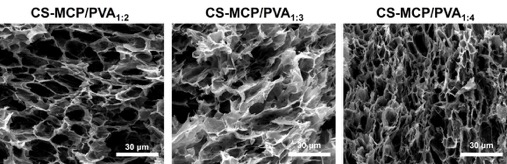


**Figure S3.** Structural and morphological characterization of hydrogels.


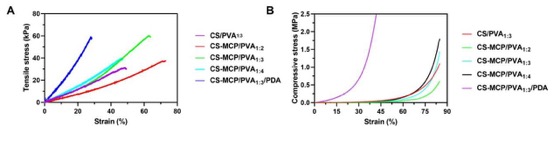


**Figure S4.** Tensile stress (A) and compression stress (B) of different hydrogels.

**Figure S5.** Swelling ratio of hydrogels (data are represented as mean ± standard deviation (SD), n = 3).


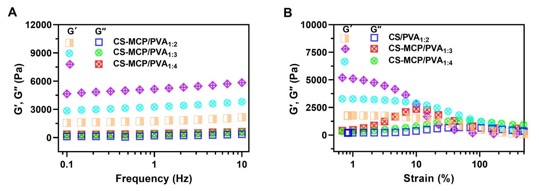


**Figure S6.** Rheological properties of hydrogels: The G′ and G″ of hydrogels with changing frequency and strain.


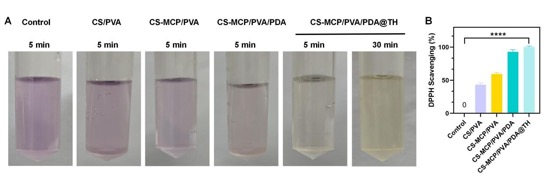


**Figure S7.** (A) Photos of DPPH solutions at different time points and DPPH solutions treated with various hydrogels. (B) DPPH clearance rate (Data are means ± SD, n = 3; *****p* <0.0001).


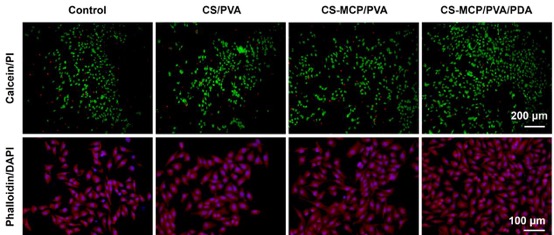


**Figure S8.** Live/Dead cell staining and cytoskeleton staining of HUVECs after 24 h.


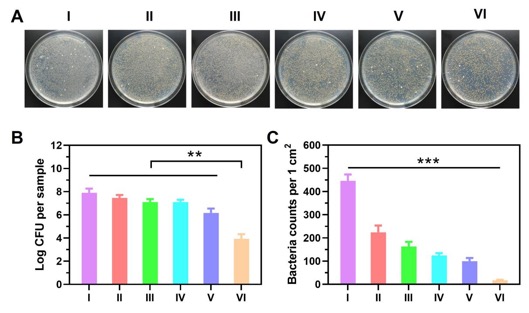


**Figure S9.** (A) Residual bacteria were obtained from the wound tissues and cultured on LB agar plates. (B) Total bacterial CFU (log) on the LB agar plates. Group I represents the initial *S. aureus* count. (C) Quantitative analysis of bacteria counts per 1 cm^2^ on LB agar plates. (Ⅰ: Control; Ⅱ: CS/PVA hydrogel; Ⅲ: CS-MCP/PVA hydrogel; Ⅳ: CS-MCP/PVA/PDA hydrogel+NIR; Ⅴ: CS-MCP/PVA/PDA@TH hydrogel; Ⅵ: CS-MCP/PVA/PDA@TH hydrogel+NIR; data are represented as mean ± SD, n = 3, ***p* < 0.01, ****p* < 0.001)


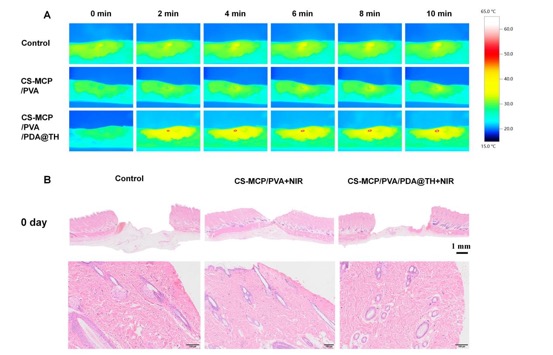


**Figure S10.** (A) Thermal imaging during the treatment process. (B) H&E staining of the infectious wound tissue 0 day after treatment. Scale bar: 1 mm, 100 μm.


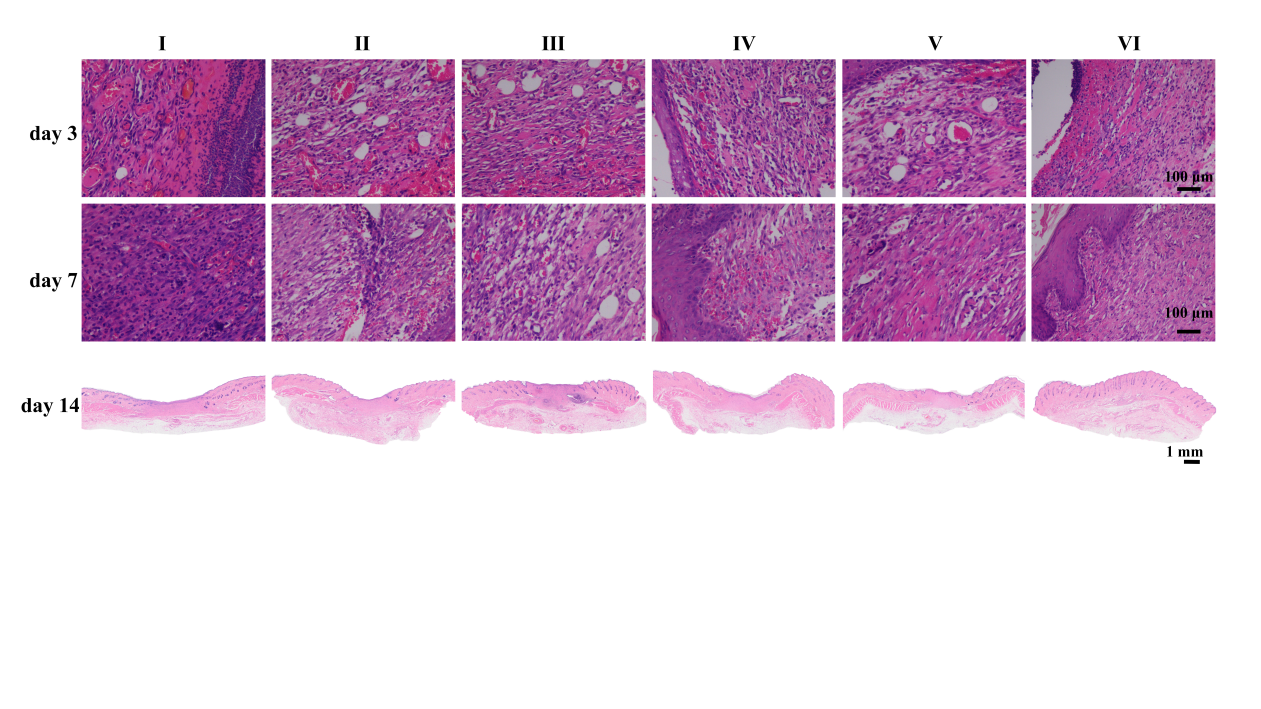


**Figure S11.** H&E staining of the infectious wound tissue on day 3, day 7 and 14 day after treatment (Ⅰ: Control; Ⅱ: CS/PVA hydrogel; Ⅲ: CS-MCP/PVA hydrogel; Ⅳ: CS-MCP/PVA/PDA hydrogel+NIR; Ⅴ: CS-MCP/PVA/PDA@TH hydrogel; Ⅵ: CS-MCP/PVA/PDA@TH hydrogel+NIR). Scale bar: 100 μm, 1 mm.


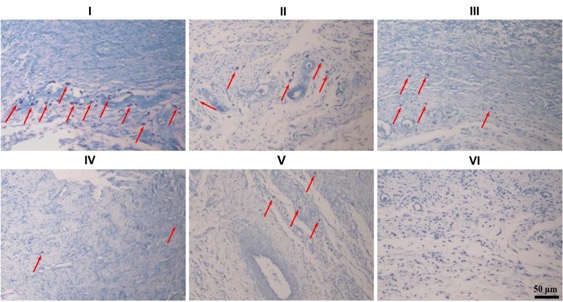


**Figure S12.** Giemsa staining of the infectious wound tissue on day 3 after treatment (Ⅰ: Control; Ⅱ: CS/PVA hydrogel; Ⅲ: CS-MCP/PVA hydrogel; Ⅳ: CS-MCP/PVA/PDA hydrogel+NIR; Ⅴ: CS-MCP/PVA/PDA@TH hydrogel; Ⅵ: CS-MCP/PVA/PDA@TH hydrogel+NIR). Red arrows indicate the residual bacteria. Scale bar: 50 μm.


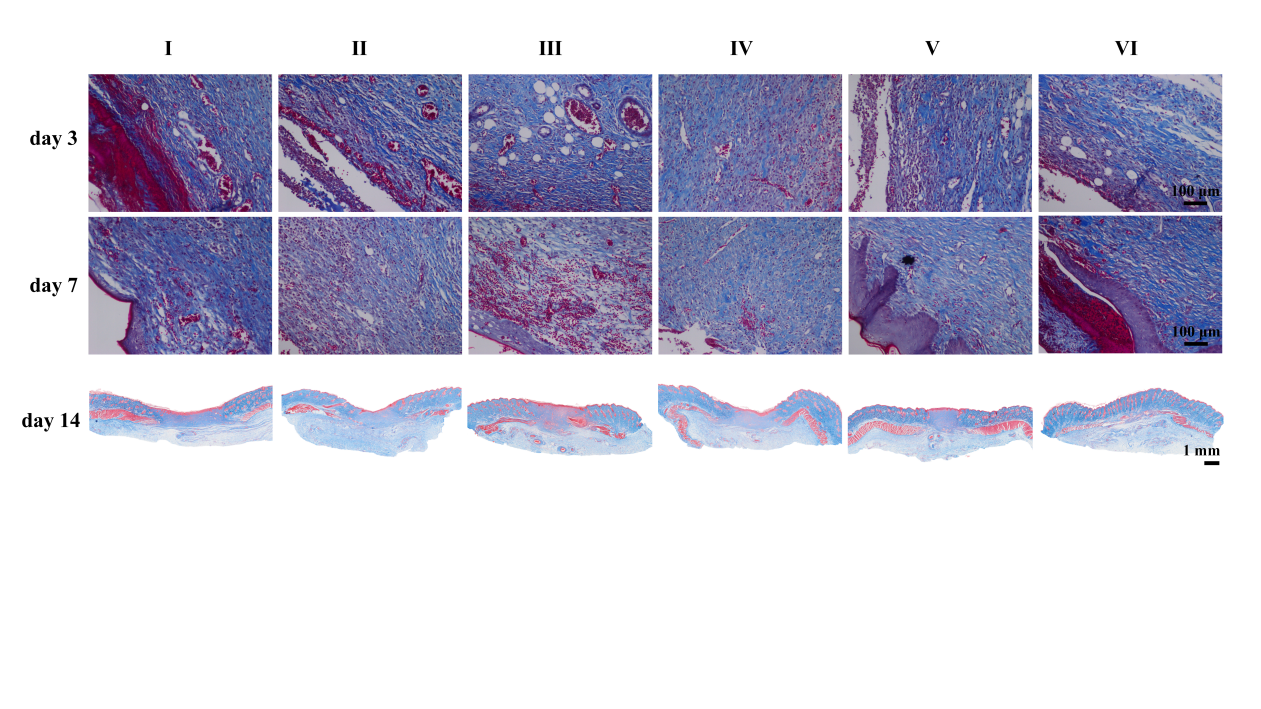


**Figure S13.** Masson’s trichrome staining staining of the infectious wound tissue on day 3, day 7 and 14 day after treatment (Ⅰ: Control; Ⅱ: CS/PVA hydrogel; Ⅲ: CS-MCP/PVA hydrogel; Ⅳ: CS-MCP/PVA/PDA hydrogel+NIR; Ⅴ: CS-MCP/PVA/PDA@TH hydrogel; Ⅵ: CS-MCP/PVA/PDA@TH hydrogel+NIR). Scale bar: 100 μm, 1 mm.


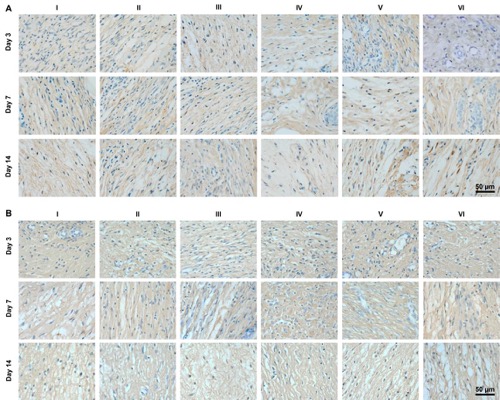


**Figure S14.** Immunohistochemical staining analysis for (A) collagen I and (B) collagen III expression in wounds treated by hydrogels (Ⅰ: Control; Ⅱ: CS/PVA hydrogel; Ⅲ: CS-MCP/PVA hydrogel; Ⅳ: CS-MCP/PVA/PDA hydrogel+NIR; Ⅴ: CS-MCP/PVA/PDA@TH hydrogel; Ⅵ: CS-MCP/PVA/PDA@TH hydrogel+NIR). Scale bar: 50 μm.


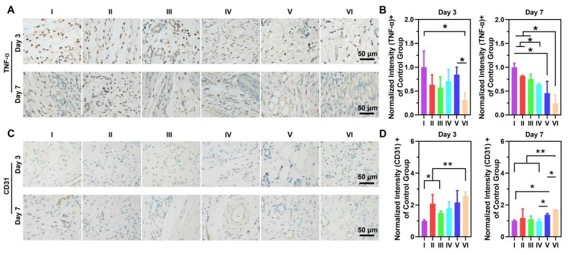


**Figure S15.** Immunohistochemical staining analysis for TNF-α and CD31 on day 3 and day 7 (Ⅰ: Control; Ⅱ: CS/PVA hydrogel; Ⅲ: CS-MCP/PVA hydrogel; Ⅳ: CS-MCP/PVA/PDA hydrogel+NIR; Ⅴ: CS-MCP/PVA/PDA@TH hydrogel; Ⅵ: CS-MCP/PVA/PDA@TH hydrogel+NIR). A) Immunohistochemical staining of TNF-α. B) The relevant quantitative analysis of TNF-α. C) Immunohistochemical staining of CD31 expression. D) The relevant quantitative analysis of CD31. Scale bar: 50 μm; **p* < 0.05, ***p* < 0.01.


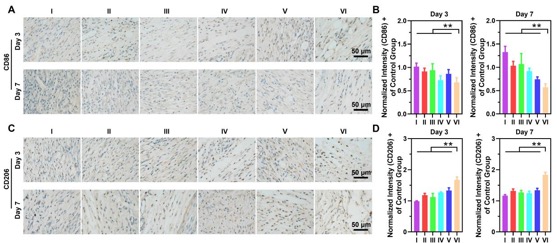


**Figure S16.** Immunohistochemical staining analysis for CD86 and CD206 on day 3 and day 7 (Ⅰ: Control; Ⅱ: CS/PVA hydrogel; Ⅲ: CS-MCP/PVA hydrogel; Ⅳ: CS-MCP/PVA/PDA hydrogel+NIR; Ⅴ: CS-MCP/PVA/PDA@TH hydrogel; Ⅵ: CS-MCP/PVA/PDA@TH hydrogel+NIR). A) Immunohistochemical staining of CD86. B) The relevant quantitative analysis of CD86. C) Immunohistochemical staining of CD206 expression. D) The relevant quantitative analysis of CD206. Scale bar: 50 μm; ***p* < 0.01.
